# Items in Brief

**Published:** 1873-08

**Authors:** 


### Items in Brief.

—Puerperal Fever has been so prevalent in New York City of late as to compel certain of the practitioners there, the *Record* says, to send their patients out of town to be confined.—The South Brooklyn Dispensary was changed by recent act of Legislature to the Southern Dispensary and Hospital. Dr. G. W. Friou, surgeon to the institution favors us with an article this month on Electricity.——Dr. J. J. Caldwell of Baltimore, has kindly sent us a copy of the Constitution and By-Laws of the Maryland Epidemilogical Society. This

Association embraces some of the most active practitioners of Baltimore and vicinity, and its meetings are largely attended.——On Hospital Sunday in London, from £20,000 to £30,000 was contributed in the various churches in the city for the support of the city Hospitals. Could not some such plan be introduced in Buffalo?——Dr. S. W. Butler is rapidly sending out circulars calling for information for the forthcoming Medical Register and Directory of the United States. It is desirable that the names of all physicians be included in this Directory, and medical men will confer a favor upon the publisher by forwarding their addresses at once to Dr. Butler, 115 S.Seventh St., Philadelphia. ——According to the *Philadelphia Medical Times*, Dr. Martin, Prof. of Theory and Practice of Medicine in Hahnemann Medical College in that city recommends "where no indication for special medication is found" that nux vomica be given to colored people "with reasonable assurance of success." We are left in the dark regarding the size of the dose or the kind of success implied. ——Dr. Milton Jay, Prof. of Surgery, in the Bennett Eclectic Medical College, Chicago, reports a case of Fracture of the Femur (Chicago Med. Times) in which, after being treated twenty-nine days by extension, he found by actual measurement two inches elongation. It seems to us as though there was some stretching some where, either in the leg or the story.——In a minute examination of the morbid anatomy of the sympathetic in syphilitic patients Dr. Petrow found, in twelve cases which he examined (*Virchow's Archiv.*) thickening (hyperplastic process) of the connective tissue frame-work; and changes in the ganglion-cells. These consisted in abnormal pigmentation, in colloid change, and in proliferation and enlargement of the epithelial layer surrounding the cells.——Dr. Wheeler (*Boston Med. Journal*, May '73,) reports a case of fatal vaccination, of a man with a crust taken from the arm of the patient's child. He arrives at the conclusion that vaccination from the crust was more dangerous than from lymph.

PERSONAL.—Dr. Burrows has been re-elected President of the Royal College of Physicians of London, an office which he has filled for the last four years. —*Record.*——Dr. Mary L. Wadsworth, a graduate of Mount Holyoke Seminary and a former practitioner in Springfield, Mass., is now family physician to the Sultan of Turkey.—*Record.*——Prof. Chas. L. Ives of the Medical Department of Yale College, has been elected to fill the chair of diseases of the mind and nervous system in the Medical Department of the University of New York City. Dr. David P. Smith of Springfield, Mass., has been nominated for the professorship of Theory and Practice of Medicine, lately occupied by Prof. Ives.——Dr. Tyler Smith, the well known Obstetrician died from the effects of Cerebral Hemorrhage on the 3d. ult. Dr. Smith has been in ill health for some time and symptoms of Brights Disease and arterial degeneration had been detected. As a writer on Obstetrics, Dr. Smith had a wide reputation.

